# Young Adults’ Perspectives on the Implications of an Augmented Reality Mobile Game for Communities’ Public Health: A Qualitative Study

**DOI:** 10.3389/ijph.2023.1605630

**Published:** 2023-03-01

**Authors:** Yuk Chiu Yip, Ka Huen Yip, Wai King Tsui

**Affiliations:** ^1^ Li Ka Shing School of Professional and Continuing Education, Hong Kong Metropolitan University, Hong Kong, Hong Kong SAR, China; ^2^ School of Health Sciences, Caritas Institute of Higher Education, Hong Kong, Hong Kong SAR, China

**Keywords:** qualitative, location-based games, gaming motivation, young adults, gaming experiences

## Abstract

**Objectives:** Several physical, psychological, and social health consequences are caused by smartphone users’ addiction to games. A location-based game (LBG), Pokémon GO, recently garnered significant attention from young people. This study aims to explore their experiences with this game and motivations for playing, investigating their perspectives on the game’s implications for themselves and the public health of their communities.

**Methods:** Ten qualitative focus group interviews were conducted. Young adults, aged 18–25 years (*n* = 60), were recruited in Hong Kong. Data were analyzed using a thematic approach.

**Results:** Five themes emerged: 1) missing out or self-regulation, 2) childhood memories of Pokémon, 3) extending virtual-reality exploration, 4) spending more time outdoors walking and exercising, and 5) getting together with others and social interaction.

**Conclusions:** This study showcases the motivational factors of young adults and their cohorts in societies worldwide. LBGs may impact players’ physical and social activity levels, and behavior. Nonetheless, certain negatives were identified (i.e., addiction and behavior resulting from a loss of self-control). These negatives deserve health practitioners’ attention and future studies should explore possible public health interventions

## Introduction

The last decade witnessed an emergence of the prevalent use of smartphones. Such devices have become a vital component of daily life, and their integration has been facilitated by their low cost and technological advances. Smartphones with sophisticated mobile operating systems have characteristics similar to those of handheld personal computer systems (1). In Hong Kong (HK), the demographic that mostly uses and owns smartphones involves young people, with nearly 92% over the age of 10 (2). Internet services, online entertainment, games, telecommunications, and social connections are all possible *via* multifunctional smartphone devices (3–8). The psychological and physical health of smartphone users who play online games remains an active area of public health research (9–15).

Location-based games (LBGs), a mobile technology enabling people to experience immersive game environments, provides augmented reality environments in which digital images are layered onto reality (16, 17). Pokémon GO is an augmented reality game with an LBG component (4, 18, 19). Players participate in a virtual environment through their smartphones, in which they hunt for and catch digitally animated characters. Real geographical locations, tied to virtual environments, are a distinctive characteristic of LBGs. Various animated characters are displayed in real-world locations *via* augmented reality, which draws in young users. After downloading the game or application, users search for the animated characters in the physical environment that is familiar to them, that is, their neighborhood. Thus, physical exercise in the form of walking around one’s neighborhood is stimulated and encouraged, as many people are drawn into the game and its mechanics. However, the physical and mental health of an individual might also be negatively impacted by the excessive time spent playing LBGs (3, 12, 17, 19).

Consequently, it is crucial to explore how the physical and mental wellbeing of young people are impacted by LBG engagement. In terms of the social, psychological, and physical impacts of gaming addiction, multiple effects on health are noted in the literature (7, 9, 12, 17, 19–23). On the one hand, depression can be alleviated by such engagement, and the benefits of physical exercise can mitigate Type 2 diabetes and reduce the issues caused by obesity (9, 24–29).

On the other hand, players may become addicted to games (30–35). When enthusiasm gives way to addiction, studies have shown that the health consequences may become decidedly negative (16, 36, 37). Nonetheless, individual health can be beneficially affected by games that encourage healthy lifestyles (38). Patients suffering from pain have reported the utility of LBGs in distracting them from their discomfort (4, 39). Depression, social anxiety, and other psychological disorders experienced by players can also be alleviated through game engagement (40, 41). Furthermore, the reduction of sedentary behavior and elevated physical activity are direct outcomes for anyone who engages with Pokémon GO, regardless of age (42–44). This is exemplified by the approximately 2,500 steps taken daily by LBG players, per statistics provided by location-based technology (45, 46).

Following a thorough literature review, we found that the health-related implications associated with the continual engagement in LBGs have not been studied extensively among young adults. Thus, this qualitative study aims to explore the insights of their experiences with Pokémon GO as a well-known LBG and their motivations for playing, in order to investigate their perspectives on the game’s implications on the public health of their communities. In alignment with the above-mentioned aims, the research objectives of this qualitative study are as follows: 1) to explore the underlying factors that constitute the primary driving force for young adults to develop continual engagement with LBGs—in our case, Pokémon GO, and 2) to examine the implications of the continual engagement of LBGs for the young adults in terms of their social, psychological, and physical wellbeing, and in relation to the public health of their communities at large.

## Methods

Focus groups (FG) were used to collect data from group interactions and discussions providing diverse explanations and outlooks concerning the central topics (47). This study was approved by the Committee on the Use of Human and Animal Subjects in Teaching and Research at the Tung Wah College (Ref. no. RESC2017003). Participants provided written consent for their participation in this study and were assured that their experiences and interview content would be reported anonymously in an international journal. The details of the methodological considerations were provided in Table 1.

**TABLE 1:** Methodological considerations (Hong Kong, China, 2017).

Participants	The inclusion criterion for selection as a participant encompassed being a recent or currently active player of Pokémon GO. Interest in study participation was ascertained by contacting potential participants individually; the eligibility criteria for participant selection, the objective, and methodology of the paper were communicated to the participants. The time and venue for data collection procedures, assessment of eligibility, and elucidation of the study’s objectives were discussed in interactions with potential participants. Regarding the demographic information of the participants, the occupations of these participants were not collected as the research team believed that this socio-demographic characteristic did not seem relevant to the current research’s aims and objectives. Thus, based on the ethical principle to avoid collecting unnecessary participant information, the research team decided to not include the question of occupation in the data collection process.
Data collection	Prior to the consent process, the participants were permitted to ask questions. The risks, methodology, and objectives of the study were communicated to them at the beginning. The audio of the FG sessions was recorded with the permission of the participants. The focus group questions in Table 2 were used to determine what factors in social, individual, interpersonal, and environmental domains relate to the insights of young people’s experiences with Pokémon GO, their motivations for playing, and their perspectives on the game’s implications for the public health of their communities (48, 49). The FGs were moderated by the second author (KH), who has extensive experience in administering qualitative health research techniques. To ensure that the questions, the flow of the discussion, and the content were well received, a pilot FG was conducted by the research team (YC, KH, and WK). Viewpoints following LBG engagement and alterations in physical activity were discussed with the participants. The discussion ended after further details and interpretations were sought through additional inquiries. Based on the availability of participants, FG interviews were conducted in a quiet and comfortable room of a local academic institution.
Data analysis	Codes corresponding to 1) the underlying factors that constitute the primary driving force for young adults to develop continual engagement with LBGs and 2) the implications of the continual engagement of LBGs were determined *via* an analysis of the transcripts during the initial coding phase conducted by YC and KH, thus enacting a deductive process (48, 50). Additionally, an inductive process was undertaken by WK to examine the transcripts (50). As part of the study’s analysis strategy, themes and secondary codes, in line with the ecological model, were generated following a research team discussion (YC, KH, and WK). The final coding was cross-checked by different team members. Finally, the themes, topics, and insights outlined in the FGs were subject to a final check by YC and KH, to ensure that all possible areas were covered.
Ethics statement	This study was approved by Tung Wah College, Committee on the Use of Human and Animal Subjects in Teaching and Research (HASC) (Ref. no. RESC2017003). All participants provided written consent for their participation in this study. All participants were assured that the experiences they share, and their interview content would be reported in an international journal anonymously.

### Participants

The snowball sampling technique was used to recruit 18- to 25-year-old participants. In this study, a total of 60 participants were recruited. A large proportion (75%) of the participants included males, although the applied sampling strategy did not preclude the participation of female participants. The average age of the participants was 20.9 years.

### Data Collection

Participant recruitment and data collection were conducted by YC, KH, and WK. Between July 2017 and July 2018, ten FG discussions were conducted. Each discussion lasted approximately 45–60 min, consisting of five to seven participants per group. The basic demographic information of the participants (such as the age and sex) and the participants’ activity on Pokémon GO were collected prior to the commencement of the FG.

A literature review was performed by the research team (YC, KH, and WK) on online games and LBGs, including the psychosocial and physical health perceptions linked to gaming. The review served as the basis for the discussion guide formulated in this paper, as shown in Table 2 (4, 23, 51–54). Data saturation was confirmed by all researchers at the completion of the eighth FG discussion, with no novel data arising from subsequent rounds of the FG (55).

**TABLE 2:** Focus group discussion guide (Hong Kong, China, 2017).

-	Can you describe your experiences of playing Pokémon GO outside?
-	Since the game launched, what do you think its impact on daily life has been from a public health perspective?
-	What attributes of the game do you find most appealing?
-	How has your social life changed since you began playing Pokémon GO?
-	From a public health perspective, how do you think the game alters your interactions with friends, family, and strangers?
-	From a third-person perspective, can you share how your family and acquaintances rate the Pokémon GO game? What do you think about their opinions?
-	If there were no Pokémon GO now, how would you use the time that you used to devoted to it?
-	Overall, based on your perceptions of public health, what do you think are the negatives and positives of playing Pokémon GO?

### Data Analysis

The thematic analysis method was used to examine the verbatim transcriptions of the audio recordings of the FG sessions (50). Initial coding and code refinement of the transcripts were facilitated using the NVivo 12 qualitative software (QSR International, Melbourne, Australia). The themes ultimately derived from the analysis process were discussed in the results section.

## Results

As Table 3 illustrates, the Pokémon GO trainer level of the participants was as follows: two were between trainer levels 16–20; four between trainer levels 21–25; nine between trainer levels 26–30; nine between trainer levels 31–35; 25 between trainer levels 36–40; and 11 above trainer level 41. Table 3 also illustrates the amounts of money the participants spent on Pokémon GO as at the interview day: nine spent no money; three spent between 1 and 12 USD; nine spent between 13 and 64 USD; 27 spent between 65 and 130 USD, and 12 spent over 130 USD. The average playing time, per day, was 4.9 h and the average duration spent playing was 5.3 months.

**TABLE 3:** Descriptive data of the study participants and their activity on Pokémon GO (*N* = 60) (Hong Kong, China, 2017).

Measure	n	Percentage (%)
Sex (Number of participants)		
Female	15	25
Male	45	75
Age (Number of participants)		
18–19	12	20
20–21	24	40
22–23	15	25
24–25	9	15
Number of months spent playing Pokémon GO		
1	0	0
2	3	5
3	6	10
4	6	10
5	18	30
>6	27	45
Average number of hours spent per day		
<1	0	0
1–2	6	10
3–4	12	20
5–6	22	36.7
>6	20	33.3
Trainer level (in the Pokémon GO)		
1–5	0	0
6–10	0	0
11–15	0	0
16–20	2	3.3
21–25	4	6.7
26–30	9	15
31–35	9	15
36–40	25	41.7
>41	11	18.3
Money spent (USD) as at the interview day		
0	9	15
1–12	3	5
13–64	9	15
65–130	27	45
>130	12	20
Number of venues for playing		
1	0	0
2	0	0
3	0	0
4	3	5
5	3	5
6	21	35
All locations	33	55

Note: There were several venues for playing Pokémon GO, including at home, at school, in a shopping mall, in a park, at work, while traveling, and all locations.

### Theme 1: Missing Out or Self-Regulation

Addiction, harming oneself or others by colliding with objects on the street, or social disturbance were among the potentially harmful impacts that every participant acknowledged as a possibility resulting from playing LBGs.

Like in Morse Park, when many players rushed to the park to hunt for new species of Pokémon. But there were some people there playing football, and they [the football players] did not know that the [Pokémon GO] players were rushing into the park. So, they ran into one another (19-year-old male participant).

Many participants reported that they had had similar experiences as they reflected on their memories of accidents that had occurred when players were focusing on hunting for Pokémon. They recognized that players engrossed in the game in public might disturb others.

I crossed the road without looking … bumping into something or somebody or taking a wrong step (22-year-old female participant).

A lot of people blocked the road because of the appearance of Gyarados [a Pokémon character in the game that, to the participant, was rare to see]. At the time, I felt that they really seemed to create an obstruction [to the traffic] (18-year-old male participant).

There were some complaints about disturbing others while they were jogging, as the joggers claimed that there would be nowhere for them to jog if many people were jammed in there [the jogging path at Morse Park] (24-year-old male participant).

Some conflicting opinions were expressed concerning players’ lack of self-control. Apart from mobility and improved sociability, participants who were loyal fans of Pokémon GO, devoted a huge sum of their pocket money to make in-game purchases. Participants agreed that, once an individual demonstrated behavior that reflected a compromise in self-control (an excessive devotion of time to the game at the expense of sleep and rest time, an urge to devote money to “equip” the game character in order to dominate in the game, etc.), it would indicate the start of an “addiction.”

It was addictive. When you are compared with others [players], you began to lose common sense and tried to win over by collecting more rare tools and Pokémon characters in the game. Then, you were willing to spend more time, energy, and money as well for more rewarding achievements in the game [turning his head laterally to seemingly indicate this is no good in terms of self-control] (20-year-old female participant).

A lot of money was spent compared with other games. At that time, three thousand dollars [3,000 USD] was swiftly spent like pouring water from a bucket; one splash, and it was a lot of water. Also, lots of time was spent on the street; therefore, energy was lacking to do other things. The state of exhaustion made me fall asleep as soon as I got home (23-year-old male participant).

Some of them also reflected that this uncontrollable behavior affected their job performance.

We have been too focused on this game; obviously, our work efficiency has been declining (23-year-old male participant).

Some might say it is too addictive or leads to improper time management, but I think that anyone can make their own adjustments. For me, playing a cumulative sum of 8 h a day [nodding head] is a reasonable time. I think my time management is fine with the game (22-year-old male participant).

### Theme 2: Childhood Memories of Pokémon

According to the participants, the nostalgia elicited by the connection of childhood memories, from watching the Pokémon TV show and movies, to playing the LBG in reality, was the principal appeal. Their smartphone devices enabled virtual participation in the Pokémon world, but the pursuit of the Pokémon occurs physically in the real world; combined with nostalgia, this provides a pleasurable experience. Given that Pokémon was one of the most popular television series during the participants’ childhood, it is an attractive and exciting proposition. Half of the participants were inspired to begin playing Pokémon GO for this reason.

Pokémon holds a special place in my heart (22-year-old female participant).

While you’re playing Pokémon GO, you’re required to assume the first-person perspective of the game … when you are walking to a particular location. Then, a Pokémon appears in front of you. At that moment, you seem to be Ash Ketchum [main character of Pokémon] in the Pokémon movie (25-year-old male participant).

Many participants felt that the Pokémon were actually living in the real world, as the digital image of the character appeared in the setting where they were standing when the smartphone camera was on; they would then be required, in the virtual world, to throw a ball at the image to catch it. This interactive gaming experience represents a secondary factor, which motivates the participants to devote time and energy to the game, because they described it as “like I am the real character, as in the game when the character is adventuring, and you do not know what will happen next.” The surprise that comes with the adventure drives participants to explore the world in the LBG by walking around in the real world.

Compared with other games, it’s more realistic and closer to reality. When you’re playing, you cannot achieve anything or do anything else but Pokémon GO. It is like a life which is unpredictable, and surprises come without notice. This adventure-like gaming experience just deeply attracts me [repeated two times the word deeply] (23-year-old male participant).

### Theme 3: Extending Virtual-Reality Exploration

According to several participants, reality and the virtual world are connected when playing Pokémon GO. Searching HK within one’s neighborhood is incentivized for Pokémon GO players. Legendary Pokémon characters reside only in specific famous locations around the world. Consequently, the game encourages traveling to other countries or new locations nearby. At times, health campaigns and activities may be noted by the participants, increasing their awareness of some population health issues.

I hang out more around the neighborhood because of Pokémon GO. I discovered a place that I did not know about from a picture of the Pokéstop and discovered that it’s just near my house. On the way to a new location, I look around and note some health fairs (21-year-old female participant).

Some participants were active players, who expressed the enjoyment they received from hunting for Pokémon in a variety of places, while aiming to be one of the world’s master trainers. They emphasized that doing so could be a useful tool for acquiring an in-depth understanding of the geography and culture of their neighborhood.

I visited Star Ferry Pier, Laguna Park, and Victoria Park and familiarized myself with places that surrounded my destinations. Thus, I focused not only on the nature of the game itself but on going outside more and finding out more about my neighborhood. I did not notice there is an influenza vaccination promotion campaign in the park (24-year-old male participant).

### Theme 4: Spending More Time Outdoors Walking and Exercising

According to every participant, the objective of seeking out Pokémon GO’s characters tangibly altered their daily routines, modifying their walking habits and increasing the amount of exercise they undertook. The majority of participants considered it possible that people who were socially withdrawn could, in a certain sense, be brought out of their shells by Pokémon GO. A positive reaction from an autistic child was witnessed by one of the participants, who noted the child’s joy at acquiring a new character after stepping out the front door with her mother.

I think the attractive point is that it requires you to play outside, whereas for most of gaming, you usually stay at home. But this game forces you to go somewhere to hunt for Pokémon (19-year-old female participant).

I cannot hatch the eggs while taking public transportation. It’d be difficult to detect new Pokémon, so I chose to walk … I really enjoy running with my friends. We always rush after an unknown Pokémon that is available for an extremely limited time. And it made me sweat all over, including my hands, forearms, and even my underarms (22-year-old male participant).

Some of the participants experienced difficulties in altering their daily habits. Acquiring a Pokéstop or a Pokémon egg, was achieved by many participants through small alterations to their routines, such as walking to certain locations or getting off one stop earlier on their public transportation route. Particularly for age groups that were frequently physically inactive, such as the parents of the participants, exercise was promoted by the LBG engagement.

Take my family as an example. My parents would go outside too, which was really shocking. I’ve never seen my dad do something like that. There were many other middle-aged people on the street. Despite their intention to just play the game, it turned out that they exercised a lot more (23-year-old male participant).

### Theme 5: Getting Together With Others and Social Interaction

Inter-generational connection, increased peer interactions, the congregating of like-minded people, and increased social interaction are among the game’s benefits, owing to the common objective shared by all players.

Although I would seldom chat with other people, if the question “Oh! Do you play Pokémon GO?” gets asked, the topic might continue by counting the number of Pokémon [each has], hunting methods, and so on (23-year-old male participant).

I met him [one participant] only because of this game, and we always go for gym battles afterward (21-year-old female participant).

All participants expressed that there was a common goal whenever one was playing the game and that large groups of people would never miss the opportunity to hunt for a Pokémon.

Suddenly, a kid shouted that he had caught Ivysaur [a Pokémon character]. Afterward, [sic] everyone nearby took out their phones to catch it at the same time … When everyone is playing, it seems you ought to follow the trend (22-year-old male participant).

Most participants (nearly 91%) observed that they shared a common goal, which favored meeting up with or reuniting with others. They felt that the game had the potential to improve their social skills, to make them better at communicating with others.

If I’m studying nursing and they’re studying engineering, I’ll have no idea what they’re talking about when they talk about mechanics. However, Pokémon GO can be a topic that we share (23-year-old female participant).

Normally, you and your friends do not meet every night for dinner and stuff, but the game gave us the chance to say, “Hey, want to catch some Pokémon tonight?” “Sure, let’s go together” (18-year-old male participant).

Most of them (approximately 86.6%) also noted that a variety of age groups participated in this game. The common goal generated communication and interaction between different generations.

I felt that I communicated more with family, as I seldom talked to my mom when playing computer games. Sometimes, when I returned home after playing Pokémon GO, my grandpa, who was playing the game, would ask, “Where did you catch the Pokémon?” (24-year-old female participant).

## Discussion

The cognitive, psychological, and physical health of Pokémon GO players may be positively affected in their day-to-day lives by playing the LBG (56). For positive outcomes to occur in these aspects of health, the level of players’ engagement in a particular LBG over time is an important question for researchers who intend to apply LBG as a health promotion tool at a population level. Though the nature of this study as a qualitative inquiry may limit our ability to answer that important question, our study revealed that the extent of the players’ engagement and motivation to play the game is largely determined by their childhood memories (as Pokémon in this case). This finding implicates that, if a LBG is to be constructed for the purposes of health promotion, a careful selection of games (and/or the characters in games) may be required; these games and/or characters should be able to stimulate players’ memories and thereby internally motivating the players to engage in the gaming activities over time. To confirm this argument, the comments from 51 participants in our study may be relevant. They noted repeatedly in the interviews that the “mental connection [that is, how a player of LBG perceives and experiences the game]” plays a major role in maintaining the engagement in the gaming activities through time. Our study found that the gaming design of Pokémon GO enables players to feel the experiences and sentiments of an adventurous and interactive journey *via* augmented reality technology. As the participants experienced the sensation of becoming a Pokémon trainer in real life, specific memories are generated. These memories serve as a driving force to keep them engaging in the game, leading to alterations in cognitive behaviors through time (for example, an increase in the player’s social interactions with individuals in their neighborhood). Our study confirms the findings from similar research that the positive influence on mental and physical health appeared to take effect through various engaging features of LBG, namely, human interaction, community integration, and physical participation (38).

Our study findings suggested that walking more than usual was incentivized, the formation of exercising habits was stimulated, and prolonged game engagement can lead to high levels of daily physical exercise. For example, the physical activities such as walking, and running are the core of the treasure-hunt tasks required by the players in Pokémon GO. In the game, encountering other characters or stimulating the hatching of eggs, is more likely when walking than when using various forms of transportation, which led to some participants increasing the amount they walked (57). Similar to our findings, research in other countries also pointed out that, after playing the game, an average increase of between 1,000 and 2,000 steps was observed by the players (58, 59). To support the notion that exercising habits were stimulated through gaming experiences, a majority of the participants in our study mentioned that when searching for a Pokémon that was only available for a limited time, they would run toward the target. They also observed that many players develop this habit of running in their rapid pursuit of rare Pokémon characters. As pointed out in the literature (60), the increases in physical activity of an engaging player may range from moderate to substantial. However, as a word of caution, our research team also noted the drawbacks of excessive engagement of LBG using smartphones (38, 61), and these findings should be considered when LBG is used for the purposes of health promotion.

An important finding of this paper is that the application of an LBG may help integrate a community, which may have implications when LBG is used to promote health at a population level. The design and objective of LBG technology is in part conveyed through the treasure-hunt mechanic, which led the players (and the participants in our study) to explore novel locations within their communities (4, 60, 62, 63). Specifically, owing to the game requirement to search for Pokémon characters in multiple locations, participants reported an increased desire to visit places that they never or rarely would have. Increased knowledge and familiarity with one’s neighborhood or city are encouraged *via* the repeated walks through the community. As a result, approximately 80% of our participants noted that, because of their perceived closeness with the community, they are more aware of the health promotional campaigns in their cities. Other studies also indicate the effects of LBG in terms of community integration (4, 64).

Pokémon GO enables the possibility of fortifying and building human relationships, implicating that positive effects on the population health may not solely at the physical level, but also at a psychosocial level. Our study confirms that rather than electronic communication *via* text, in-person social interactions are incentivized *via* the gameplay mode (3, 4, 65). Similar to other studies (3, 66), our study found that participants engage with a common goal without social anxiety when they are virtually battling in gyms in the same geolocation. In pursuit of this common goal, players unknown to one another can communicate with each other if they are in the same vicinity.

The recent surge in Pokémon popularity has led to multiple age groups engaging with the free-to-play game (67–69). Our study reveals that players are stimulated to develop positive relationships with not only other players, but also their parents through game engagement within a family. According to the participants, common issues like generation gaps, were bridged and relationships were developed. This positive benefit on family health may be explained by that the participants’ childhood memories of Pokémon resurfaced through playing the game. Such recaptured memories are not merely enjoyed again but are lived out and reflected through playing among people of different generations, leading to the closing of communication gaps between generations (70–72).

Nonetheless, our study also highlighted one possible drawback of the application of LBG in promoting activity levels. Social disturbance and other risks within the community have been caused by the significant influx of players pouring into the streets, while being focused on the gameplay on their smartphones. Thus, players blindly crossing the street, colliding with objects in the street, and running into people, are among the physical drawbacks that other studies have identified as a result of distracted players (4).

### Limitations

One of the limitations of this study involved its qualitative methodological nature, which does not contain any statistical tests that may help in isolating the major contributive factors motivating young adults to play LBGs. Nonetheless, in terms of achieving the research aims, our study design enabled us to explore, with open-ended questions, the in-depth feelings and emotions of the players developing continual engagement with the LBG. In this sense, the research objectives were adequately addressed, according to the research team, through authentic and detailed descriptions of the participants’ perspectives and insights within contexts. Another limitation of this study may be associated with the relative proportion of male participants. Although the sampling strategy of our study did not preclude any female participants from participating (nor did the research team impose any selection criteria on the sex of the participants), a larger proportion of male participants may have certain effects on the narratives (possibly because of the differences in gaming behaviors of males and females, if any). Nonetheless, our research team ensured that data saturation was achieved, and that during the coding process, we did not detect any significant divergence of views and perspectives in terms of the participants’ sex.

### Conclusion

Our findings shed light on the novel phenomena in terms of individual and community health underlying LBG engagement. The experiences and behavioral trends of Pokémon GO players in real-life settings were revealed. It is evident that both positive and negative impacts arising from LBG engagement exist. For example, through game engagement within a family, players are stimulated to develop positive relationships with not only other players, but also their parents. On the contrary, walking more than usual was incentivized. Players’ lack of self-control, addiction, harming oneself or others by collisions with objects on the street, or social disturbance, were the potentially harmful impacts. The findings are particularly relevant to future endeavors concerning the development of public health interventions with the use of LBG with augmented virtual technology to improve population health.
